# In vivo mutagenesis of miRNA gene families using a scalable multiplexed CRISPR/Cas9 nuclease system

**DOI:** 10.1038/srep32386

**Published:** 2016-08-30

**Authors:** Anand Narayanan, Guillermina Hill-Teran, Albertomaria Moro, Emma Ristori, Dionna M. Kasper, Christine A. Roden, Jun Lu, Stefania Nicoli

**Affiliations:** 1 Yale Cardiovascular Research Center, Section of Cardiology, Department of Internal Medicine, Yale University School of Medicine, New Haven, CT 06510, USA; 2 Department of Genetics, Yale University School of Medicine, New Haven, CT 06510, USA; 3 Yale Stem Cell Center and Yale Cancer Center, Yale University, New Haven, CT, 06520, USA

## Abstract

A large number of microRNAs (miRNAs) are grouped into families derived from the same phylogenetic ancestors. miRNAs within a family often share the same physiological functions despite differences in their primary sequences, secondary structures, or chromosomal locations. Consequently, the generation of animal models to analyze the activity of miRNA families is extremely challenging. Using zebrafish as a model system, we successfully provide experimental evidence that a large number of miRNAs can be simultaneously mutated to abrogate the activity of an entire miRNA family. We show that injection of the Cas9 nuclease and two, four, ten, and up to twenty-four multiplexed single guide RNAs (sgRNAs) can induce mutations in 90% of the miRNA genomic sequences analyzed. We performed a survey of these 45 mutations in 10 miRNA genes, analyzing the impact of our mutagenesis strategy on the processing of each miRNA both computationally and *in vivo*. Our results offer an effective approach to mutate and study the activity of miRNA families and pave the way for further analysis on the function of complex miRNA families in higher multicellular organisms.

A miRNA gene is transcribed by RNA polymerase II into a primary miRNA transcript (pri-miRNA), which folds upon itself forming a hairpin structure with a precise sequence and structural motif that is then cleaved by the RNAse enzyme Drosha and its co-factor DGCR8. The processed stem-loop intermediate, known as the precursor miRNA (pre-miRNA), is exported from the nucleus and cleaved by the RNAse enzyme Dicer in the cytosol to produce a ~22 nucleotide (nt) duplex. Upon loading of one strand of the RNA duplex into the RNA-induced silencing complex (RISC), the Argonaute protein in RISC mediates pairing between the miRNA ‘seed’ sequence (nucleotides 2–8 from the 5 prime end of the mature miRNA) and the 3 prime untranslated region (3′UTR) of the target messenger RNA (mRNA), inducing mRNA degradation and protein destabilization^1^,^2^,^3^,^4^.

miRNA regulation is an important mechanism to control the expression of numerous key genes involved in development, morphogenesis, and disease pathogenesis^5^,^6^,^7^. Consequently, there has been great interest in cataloguing the function of every miRNA *in vivo*, however, these efforts have been hindered by the complex genomic organization of miRNA genes. During the evolution of higher organisms, genomic duplication events generated miRNA families comprised of multiple miRNAs with high sequence homology and identical seed regions^8^. Local duplication events have produced clusters of miRNA genes that are often transcribed as a single RNA transcript, while non-local gene duplication events have created related miRNA genes on separate chromosomes. As a result, vertebrates such as zebrafish, mouse, and human have the greatest and most intricate repertoire of miRNA genes^8^. In fact, nearly 40% of vertebrate miRNAs belong to families. Because of this genomic complexity, it has been difficult to generate vertebrates that lack multiple miRNA family members.

Unfortunately, the current genetic approaches in vertebrates only allow for the mutagenesis of single or clustered miRNA genes, but not of miRNA families originating from non-local duplication. These technologies include classical gene ablation strategies and transcription activator-like effector nucleases (TALEN)-based strategies^9^,^10^,^11^. In previous studies using TALEN mutagenesis, one TALEN pair was designed to target a single miRNA locus, while a strategy using two TALEN pairs was used to remove miRNAs within the same RNA transcript encoding for the miR-430 miRNA family^12^. More recently, a CRISPR/Cas9 approach was successfully used to target single miRNA loci in mammalian cells^13^,^14^. In these reports, customizable single guide (sg)RNAs can recognize miRNA genomic targets in close proximity to the protospacer-adjacent motif (PAM) sequence, NGG, and generate insertions and/or deletions (indels) with mutation rates that can reach up to 75–99% with minimal off-target activity^15^,^16^,^17^,^18^. While these approaches can efficiently mutagenize miRNA loci one at a time, they have not been adapted to rapidly and comprehensively generate loss-of-function models for entire miRNA families.

Here, we optimized a scalable multiplexed CRISPR/Cas9 strategy to induce heritable loss-of-function mutations of miRNA family members. We present a comprehensive survey of mutations within pri-miRNA genes encoding 45 miRNAs. Our data showed 99% of CRISPR/Cas9 mutations alter critical sequences within each hairpin pri-miRNA structure that impairs recognition by the miRNA biogenesis machinery, and thus prevents miRNA family expression *in vivo*.

## Results

### A scalable multiplexed CRISPR/Cas9 strategy to generate zebrafish miRNA mutants

**Figure 1:**
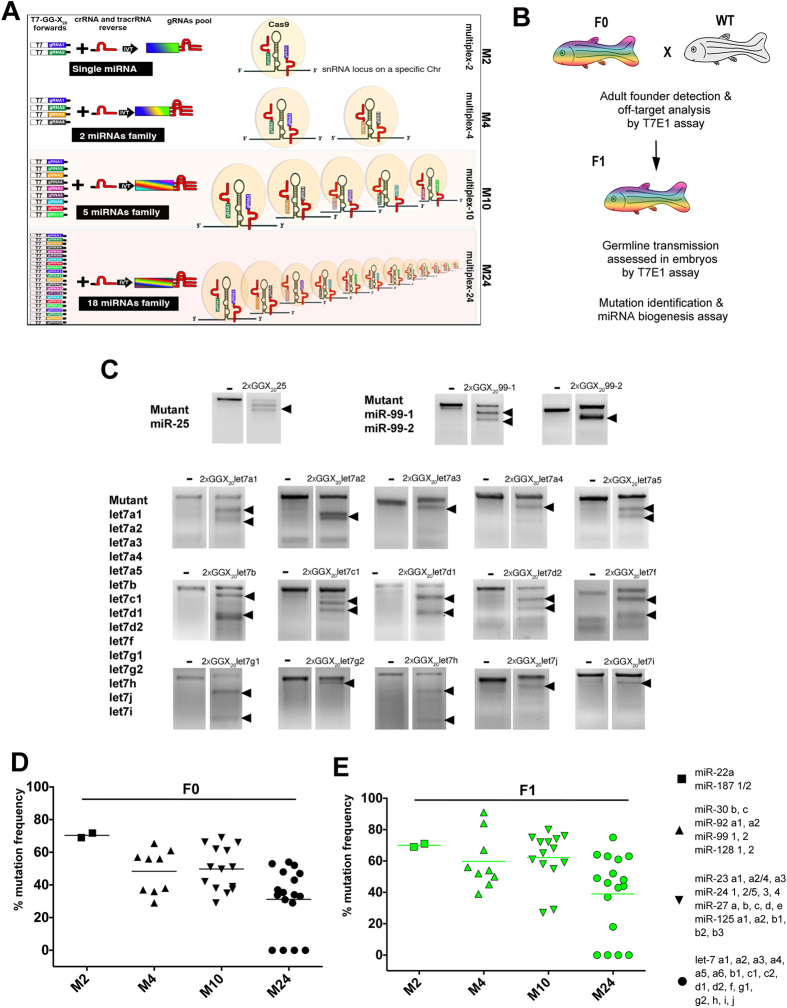
Multiplex CRISPR/Cas9 strategy to target miRNA families. (**A**) Diagram indicating the design of multiplexed sgRNAs against the miRNA family members. Pairs of forward gRNA primers were customized for each target miRNA and consisted of a 5′-T7 RNA sequence followed by GG-X_20_ (X_20_ represents the 20 nt gene specific sgRNA and the black line represents a generic 15 nt sequence for annealing with the universal reverse primer). Double-stranded DNAs containing sgRNA sequences were assembled using annealing and PCR amplification with a universal reverse primer containing crRNA and tracrRNA sequences. Pooled sgRNAs were synthetized in a single *in vitro* transcription reaction (IVT). Multiplexing (M) was accomplished to target a miRNA family and/or duplicates, consisting of 1, 2, 5, and 18 miRNAs. For the let-7 family, 24-multiplexed sgRNAs were sufficient to target 36 loci since multiple let-7 members share an identical pre-miRNA genome sequence. (**B**) Schematic representing the strategy to generate miRNA family mutants. (**C**) F0 fish injected with the respective multiplexed sgRNAs and Cas9 mRNA were genotyped using gene specific primers that amplified a 200-400 nt genomic region for one, two, and up to 18 miRNA loci. CRISPR/Cas9 mutagenesis was determined by the T7EI assay. Cleaved PCR fragments revealed the presence of indels as indicated with arrowheads. Uninjected wild type fish (minus sign) were used as negative controls. Mutation frequencies were determined by T7EI assay in genotyped F0 adult zebrafish (**D**) and F1 progeny (**E**) and quantified as previously described^19^,^22^. Each data point represents the average mutation frequency of miRNA target loci analyzed by T7E1 assay in an individual F0 adult zebrafish (**D**) or F1 embryo (**E**). At least five individual adult F0 fish (**D**) and twenty-five F1 embryos (**E**) were screened for each multiplexed sgRNA pool (M_n_).

Here, we multiplexed the injection of sgRNAs to generate mutants for miRNAs present in the genome as part of the same family, which predominantly originated from non-local duplication (Fig. 1A). First, we chose three miRNAs present as single copies and seven miRNA families as reported in miRBase v21 (Supplementary Figure 1). We tested our approach in zebrafish by targeting three miRNA families consisting of 2 miRNA members, three families consisting of 4 to 5 miRNA members, and the let-7 family consisting of 18 known members. Second, we designed two sgRNAs to target both arms of the genomic sequence of the pri-miRNA hairpin structure based on proximity to the NGG region and the mature and complementary miRNA sequences. We used the previously described 5′GG-X_20_ sgRNA architecture (where X_20_ represents the 20 nt gene specific sequence), a design that effectively discriminates off-target sites^19^. Third, we generated the DNA templates for the sgRNAs using a cloning-independent method^20^. This strategy is based on the annealing of a forward DNA oligonucleotide containing the T7 promoter sequence upstream to the GG-X_20_-N_15_, where N_15_ represents the 15 nt region that anneals with a common universal reverse primer containing crRNA and tracrRNA sequences (see Materials and Methods and ref. 21)(Fig. 1A). To generate a pool of sgRNAs that recognize many target miRNA sequences, several T7-GG-X_20_-N_15_ forward primers were added to the PCR reaction (Fig. 1A). After annealing and PCR amplification, sgRNAs were generated by T7 *in vitro* reverse transcription (IVT) and injected together with Cas9 mRNA into zebrafish embryos at the one-cell stage. F0 lesion frequency was tested for each miRNA locus in five individually injected fish using a T7 Endonuclease I assay (T7EI). Finally, we out-crossed F0 adults to generate F1 progeny and tested for miRNA indel transmission and miRNA functionality (Fig. 1B).

In the somatic tissue of individual F0 adults, we found 90% of the injected sgRNA pairs induced mutations across multiplexed conditions including 15 out of the 18 let-7 members (Fig. 1C). These targeting efficiencies were similar to a previous report showing the targeting of a single miRNA by the injection of 2 multiplexed sgRNAs led to a high targeting efficiency, close to 80% (Fig. 1D)^15^. Importantly, the progressive multiplexing of sgRNAs to target an increasing number of miRNA loci maintained a successful rate of mutagenesis for up to 24 sgRNAs (Fig. 1D). Furthermore, the analysis of F1 progeny from F0 outcrosses established that the miRNA-multiplexed mutations were heritable, demonstrating that our strategy can successfully induce miRNA mutations in the germline (Fig. 1E).

### CRISPR/Cas9 multiplexing effects on off-target sequences

**Figure 2:**
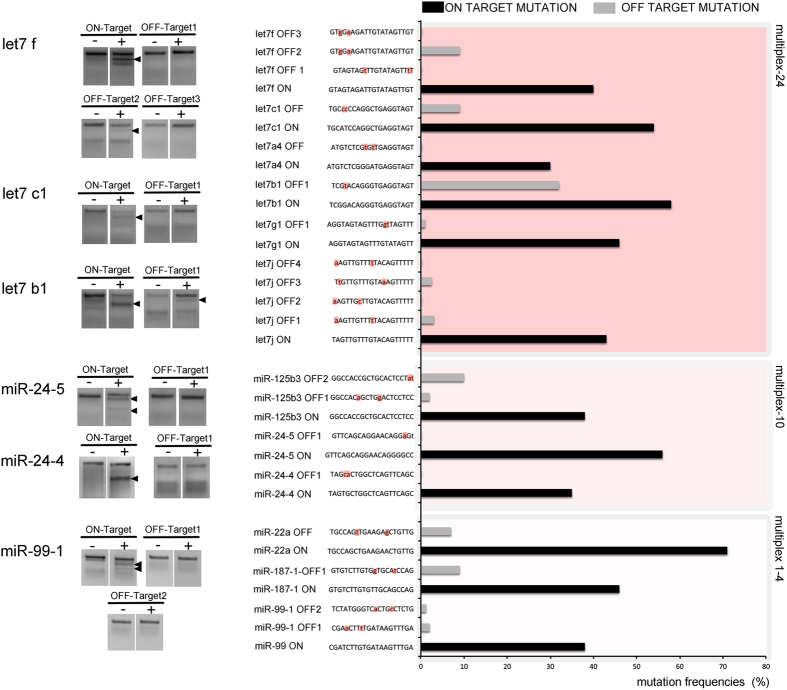
Off-target analysis of the multiplexed miRNA mutants. Off-target gene sequences were identified as previously described^19^,^24^. Off-target sequences containing 1–2 nt mismatches (indicated by a lowercase letter highlighted in red) with each of the target sgRNA sequences are shown. Off-target activity was determined using a T7EI assay after PCR amplification of the off-target loci. miRNA on-target mutations were also amplified and processed with the T7EI assay as a positive read-out of the analysis. Black arrowheads indicate mutations in both on- and off-target PCR products with a frequency ≥10%. Mutation frequencies were quantified for each on-target and off-target sequence and plotted in the chart.

Several groups have shown that CRISPR/Cas9 induces off-target mutations at sites that differ in as little as one nt compared to the target site mutations^19^,^22^. Genomic sequences encoding miRNAs share high sequence similarities, as they are critical for secondary structure formation and recognition by the miRNA biogenesis machinery^23^. Therefore, it was critical to assess the off-target effects of our mutagenesis strategy. To address this point, we first used a computational algorithm^24^ to search for sequences that have 1 or 2 nt mismatches with our target miRNA genomic sequences and thus had a high likelihood of being targeted by the sgRNAs. We then tested F0 mutant fish for these potential off-target lesions using the T7EI assay. Importantly, the frequency of off-target mutations was minimal (between 1–10%) in the majority of sequences analyzed (Fig. 2). We detected ubstantial off-target effects only in the let-7 multiplex experiment, in which one out of the eleven off-target sequences was mutated at a frequency higher than 10% (Fig. 2). Therefore, our data provide the proof-of-principle that using a high number of multiplexed sgRNAs with a 5′GG-X_20_ structure and assembled with a cloning-independent method showed high on-target efficiency with little off-target effects.

### Multiplexed miRNA mutations block miRNA family activity

**Figure 3:**
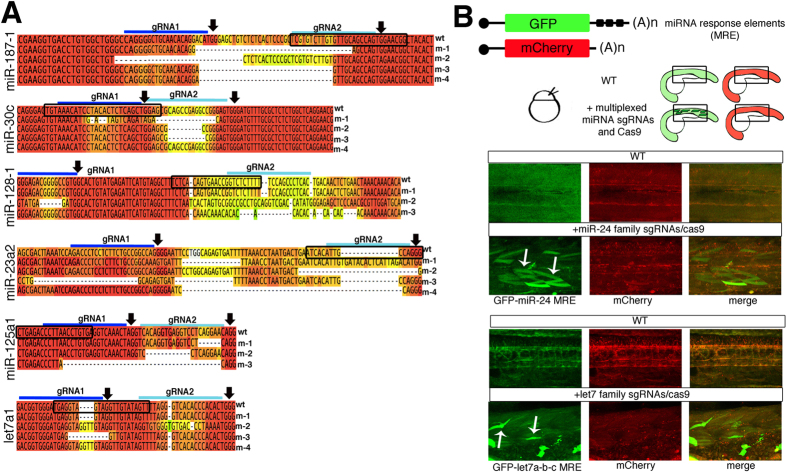
Multiplexed miRNA mutations induced efficient disruption of the miRNA family function. (**A**) Sequences of miRNA loci mutated after multiplexed CRISPR/Cas9 injection. PCR fragments were cloned and sequenced from F0 embryos. Sequences were aligned using the T-Coffee multiple sequence alignment program. The alignments are colored according to their similarity to the wild type sequence (top sequence) (high = red to low = green). The two gRNAs designed for each miRNA hairpin arm are highlighted by a solid dark blue and light blue line. Arrows point to the PAM sequence. Dashed lines indicate insertions in the wild type (wt) sequence and dashed lines in the mutant (m) sequences indicate deletions. DNA sequences within the black box correspond to the mature miRNA. (**B**) The top panel shows the diagram of the miRNA sensor assay to establish miRNA loss of function. The bottom panel shows confocal images of the lateral trunk of 48 hpf embryos injected as indicated. Caudal is to the right. Arrows indicate the presence of cells with GFP derepression in embryos after the injection of the respective multiplexed sgRNAs and Cas9.

We tested whether the induced miRNA mutations disrupted miRNA family activity *in vivo*. We cloned and sequenced 10 miRNA genomic sequences from F0 zebrafish injected with 2, 4, 10, or 24 multiplexed sgRNAs. Multiple sequence alignment analysis of sequenced PCR clones indicated that all sgRNAs induced substantial indels, independent of the multiplexing condition (Fig. 3A). Consistent with previous reports, all the mutations occurred at or near the NGG region^15^,^25^.

To test if the mutations reduced miRNA activity, we employed a miRNA gene reporter assay^26^ (Fig. 3B) in which the coding sequence for green fluorescent protein (GFP) was cloned upstream and in frame with a 3′UTR containing three miRNA responsive elements (MRE) that were perfectly complementary to miR-24 (GFP-miR-24-MRE). We chose miR-24 for the reporter assay because all miR-24 loci generate the same mature miRNA. *In vitro* transcribed GFP-miR-24-MRE and mCherry mRNAs were co-injected into one-cell stage embryos with and without the miR-24 multiplexed sgRNAs and Cas9 mixture. Although wild type GFP-miR-24-MRE positive embryos showed overall homogenous expression of the GFP and mCherry proteins, mosaic derepression of the GFP-miR-24-MRE was only observed after co-injection of the multiplexed sgRNAs-Cas9 against the four miR-24 loci (Fig. 3B, arrows). We also generated a GFP-let-7-MRE sensor by cloning three different let-7 MREs recognized by let-7 a, b and c miRNAs in the 3′UTR of the GFP reporter construct. The co-injection of GFP-let-7-MRE and mCherry mRNAs, and the M24 let-7 sgRNA pool with Cas9 induced derepression of GFP protein expression (Fig. 3B, arrows). Mosaic cells showed GFP derepression consistent with the detected indel frequency (Fig. 1D). Therefore, these data show that multiplexed miRNA mutations can significantly disrupt the activity of an entire miRNA family.

### Multiplexed mutations abrogate miRNA activity by interfering with miRNA biogenesis

**Figure 4:**
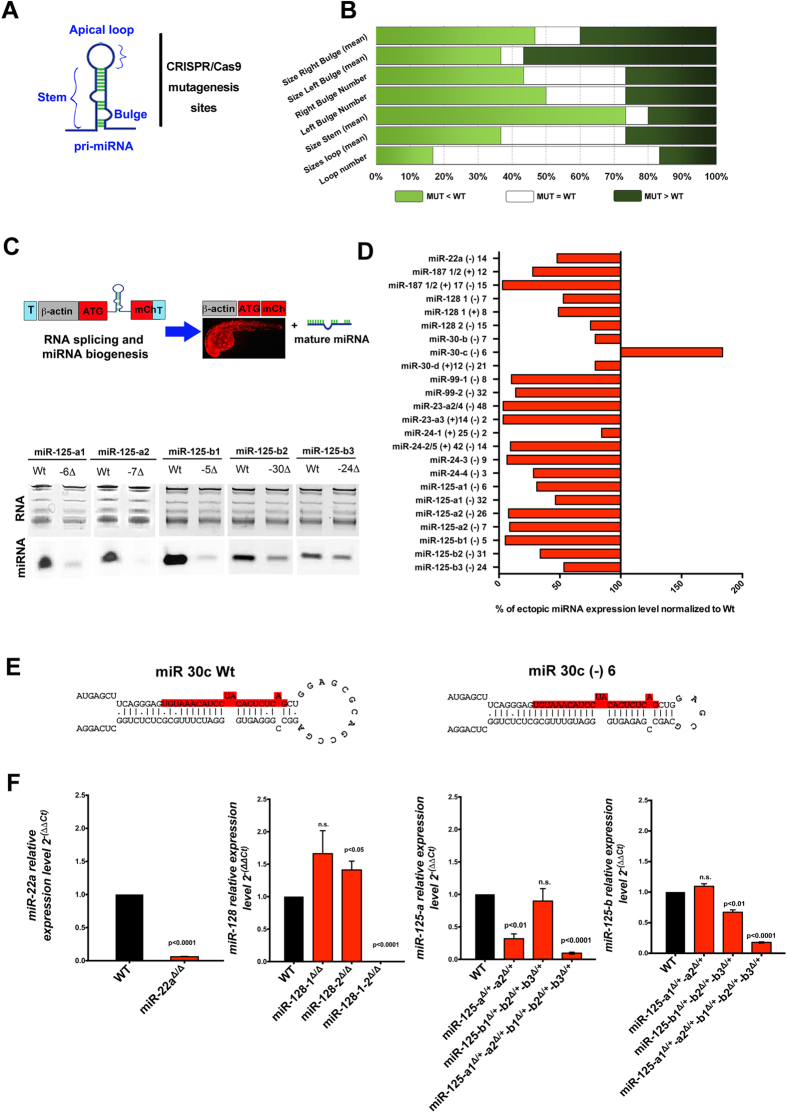
Impact of CRISPR/Cas9 mutations on miRNA processing. (**A**) Schematic representation of a pri-miRNA hairpin highlighting the position of important secondary structures and sgRNA-mediated mutagenesis sites. (**B**) Computational analysis of predicted RNA hairpin structures of wild type and mutant miRNA genome sequences analyzed. Percentile is indicative of the number of miRNA mutants falling in the indicated categories compared to the wild type structures. (**C**) Schematic representation of zebrafish miRNA biogenesis assay (top). Northern blot of miR-125 mature sequence expression obtained upon biogenesis assay of miR-125-a1, -a2, -b1, -b2 and -b3 mutants and wild type pri-miRNA genes (bottom). (**D**) Chart shows the quantification (pixel intensity) of northern blots in (**C**) normalized to the respective total RNA and the ectopic expression of wild type pri-miRNA injection. (**E**) Predicted RNA structure obtained with the ViennaRNA Software Package2 miRBase v21^36^ of the wild type and mutant sequence. Colored in red are the sequences of the respective mature miRNAs. (**F**) Average level of mature miRNA expression of the indicated miRNAs. qRT-PCRs were performed on adult fin clipped F2 miRNA founders genotyped accordingly (n = 3). Bar plots show mean + S.E.M. and significance calculations were relative to wild type embryos. n.s. (not significant, p > 0.05).

Intrigued by our results on the analysis of miR-24 and let-7 mutations, we reasoned that a comprehensive analysis of our mutagenized miRNA sequences would establish the most successful mutations to functionally perturb miRNA expression *in vivo* (Fig. 4A). We first used a computational algorithm, RNAfold^27^, to predict the maximum free energy structure of an RNA sequence and HairpIndex (Roden C. *et al.* submitted) to annotate structure and sequence features of the hairpin. Structural features such as the number and position of bulges, stem length^28^, and loop size^29^,^30^ are critical for pri-miRNA recognition and cleavage by the Drosha/DGCR8 complex. Then we compared the resulting RNA structure predictions between wild type and mutant pri-miRNA genes and identified that the majority of our insertions and deletions (indels) alter the length of the hairpin stems and the number of left bulges (Fig. 4B).

To assess the impact of mutations in the miRNA genomic sequence on miRNA biogenesis, we developed an mCherry reporter assay. We cloned the wild type or the mutant pri-miRNA sequence into an mCherry reporter construct, the pTol2-Bact-pri-miRNA-mCherry biogenesis assay vector (Fig. 4C)^31^. Upon injection of this transgenic vector in embryos, the beta-actin promoter drives the ubiquitous expression of the pri-miRNA-mCherry transcript. Following the splicing of this ectopic transcript, mCherry is expressed and the pri-miRNA is available for processing by the endogenous miRNA machinery into the mature miRNA (Fig. 4C). Using this strategy, we isolated embryos at 24 hpf that were mCherry positive and expressed wild type or mutant pri-miRNA. These embryos were processed for northern blot analysis to compare the respective mature miRNA levels between the wild type or mutant pri-miRNA. Strikingly, our quantification revealed that ~90% of the CRISPR/Cas9-induced mutations disrupted the biogenesis of ectopically expressed mutant pri-miRNAs (Fig. 4C,D). Interestingly, we found one case, miR-30c, in which mutations within this pri-miRNA gene produced an RNA hairpin that was more efficiently processed by the miRNA biogenesis machinery (Fig. 4D). Analysis of the secondary structure revealed that this mutation reduces the size of the apical loop on the RNA hairpin of miR-30c and increases stem length from 32 nt to 33 nt, thus indicating that particular modifications of the miRNA gene sequence may increase the efficiency of miRNA biogenesis (Fig. 4E). Finally, in agreement with each specific genotype, mature miRNA expression was diminished or lost in our miRNA single, double, and quintuple F2 mutant founders selected from our screening (Fig. 4F). Taken together, our data show that mutations within the miRNA gene resulted in alterations in the hairpin structure and consequently in the miRNA expression, confirming that our scalable mutagenesis strategy enables the study of miRNA family activity.

## Discussion

Here, we describe a successful strategy to multiplex the CRISPR/Cas9 system to target miRNA gene families. This method can generate *in vivo* heritable mutations in multiple miRNA loci to abrogate the activity of an entire miRNA family and enables the functional analysis of miRNA gene families in biological processes (such as development). In addition, our system can advance the understanding of the critical structural rules of a miRNA gene and how they impact miRNA expression levels.

Our strategy represents a significant improvement from the preexisting protocols. First, we used multiplexed CRISPR/Cas9 to target multiple non-coding genomic sequences in an *in vivo* model. Second, we showed that multiplexed mutations were transmittable to the F1 generation. Finally, we analyzed the impact on miRNA biogenesis of a significant number of mutations within the pri-miRNA genes. Our data showed that the multiplexing strategy simultaneously generates mutations in up to 14 miRNA loci with minimal off-target effects. Additionally, we provide the proof-of-principle that F0 and F1 miRNA family mutants can result in loss of miRNA activity. Indeed, the injection of our CRISPR/Cas9 against the let-7 superfamily or miR-24 was sufficient to disrupt mature miRNA activity leading to derepression of a GFP sensor containing miRNA-binding sites. As our mutagenesis was successfully applied to several different miRNA families, our data represent the largest collection of compound miRNA family mutants generated by CRISPR/Cas9. Our strategy enables the analysis of more complex biological questions related to miRNA function, such as the functional redundancy between miRNAs that share the same seed region, are expressed within the same cell type, or are part of the same signaling pathways. Therefore, our protocol can be applied to address critical questions related to miRNA functional complexity *in vivo*.

We analyzed the effects of a comprehensive number of indels generated in the pri-miRNA genome family sequences on miRNA biogenesis. We discovered that most of the changes affecting predicted nucleotide pairing and overall length of the pri-miRNA stem length are sufficient to impair mature miRNA biogenesis. This may be due in part to the fact that many of our sgRNAs were designed to target mature miRNA sequences within the pri-miRNA. Indeed, sequence regions and features within the stem of the pri-miRNA (bulge size, bulge position, stem length) would be preferentially disrupted by indels in this region. Interestingly, we found a mutation within a miRNA that can also enhance miRNA expression. We identified a mutation in miR-30c that reduces the size of the apical loop and increases the stem length of the pri-miRNA. Apical loop size has previously been reported to control microprocessor cleavage efficiency in mammalian cells with optimal apical loop size ranges of 3–23 nt^29^ or >10 nt^30^. The miR-30c (−) 6 mutation reduces the predicted apical loop size from 13 nt to 5 nt which may disrupt this feature. The likely mechanism of processing enhancement is the change in stem length. Optimal stem length of 35 ± 1 nt in mammalian cells has been reported to enhance microprocessor cleavage^28^. The miR-30c (−) 6 mutation increases the stem length from 32 to 33 nt which is closer to optimal stem length. Thus, further analysis in which changes in stem length and apical loop size are independently assessed will be necessary to understand this result on miR-30c biogenesis. Overall, our data showed that mutations that are not restricted to miRNA mature sequences are sufficient to alter miRNA expression. This is consistent with recent evidence provided by genome wide analysis of hundreds of human pri-miRNA gene sequences and suggests that there are evolutionarily conserved structural and sequence features that miRNA genes require for miRNA expression^14^,^28^.

Altogether, the application of our strategy will allow not only the generation of models to study miRNA family expression and function, but also the identification of novel and critical sequences within a miRNA gene essential for miRNA biogenesis *in vivo*. In light of studies showing that polymorphisms in human miRNA genes are strongly associated with disease susceptibility such as schizophrenia and diabetes^32^,^33^,^34^, strategies such as ours are critical to improve our current understanding of the impact that genetic variants have on miRNA gene sequence affecting miRNA regulation and activity.

## Experimental Procedures

### Fish husbandry

Zebrafish (strain WT^CF^)^26^ were raised and maintained according to protocols approved by the Yale University Institutional Animal Care and Use Committee (IACUC). All methods were carried out in accordance with the relevant guidelines and approved under IACUC protocol number 2015-11473.

### Generation of sgRNA, Cas9 mRNA and zebrafish injections

To generate sgRNA we purchased forward primers with the following architecture: 5′-TAATACGACTCACTATA-GG-X_20_-GTTTTAGAGCTAGAA-3′ (X corresponds to the nucleotides of the sgRNA sequence, see Supplementary Table 1) (IDT and Keck Oligos, Yale). 10 μmols of each forward primer and 50 μmols of universal reverse primer 5′-AAAAGCACCGACTCGGTGCCACTTTTTCAAGTTGAT AACGGACTAGCCTTATTTTAACTTGCTATTTCTAGCTCTAAAAC-3′ (PAGE purified from IDT) were used in an annealing and dNTP filling PCR reaction (95 °C for 3 minutes, 95 °C for 30 sec, 45 °C for 30 sec, 68 °C for 20 sec, cycle to step 2 for 34 cycles, 72 °C for 5 minutes and hold at 4 °C). The PCR product was purified using a PCR purification kit and *in vitro* transcription was performed with the T7 Flash kit. The transcript was treated with 0.5 μl DNAse Turbo at 37 °C for 20 minutes, precipitated with sodium acetate and 100% EtOH, and washed twice with 70% EtOH. Zebrafish codon optimized pT3TS:nCASn plasmid^15^ was linearized with XbaI and *in vitro* transcribed using mMESSAGE mMACHINE T3 Transcription Kit. The RNA was purified using an RNeasy Mini Kit. Zebrafish embryos at the one-cell stage were injected with 2 nanoliters of solution containing 100 ng/μl of multiplexed sgRNA with 150 ng/μl of Cas9 mRNA and Phenol Red. Higher doses of gRNA (200 ng/μl gRNA and 300 ng/μl of Cas9) yielded similar mutation efficiencies but had higher toxicity.

### Determination of on-target and off-target mutations

Genomic DNA was isolated using a DNeasy Blood and Tissue kit from a clutch of 15–20 embryos at 24 hpf injected with sgRNA and Cas9. Genomic DNA (50 ng/μl) was used to amplify an approximately 200–400 bp region surrounding the intended target (see Supplementary Table 1). Mutations were detected through a T7 endonuclease I (T7E1) assay^19^. Once the mutation was detected, the rest of the embryos from the same clutch were raised. One-month old, F0 founder fish were screened for on-target and off-target mutations through fin clipping or isolated sperm cells from individual fish using the T7EI assay or PCR Fragment Analysis. ImageJ64 software was used to quantify the T7EI digested PCR band detected in the represented agarose gels. Indels were confirmed by cloning each PCR reaction into a pGEM-T vector and sequence analysis was performed through T-Coffee Multiple Sequence Alignment^35^.

### Pri-miRNA Computational Structure Predictions

Predicted mutant and wild type pri-miRNA maximum free energy structures were predicted using RNAfold version 2.1.9^27^,^36^ and annotated using HairpIndex Matlab code (Roden *et al.* submitted). Briefly, hairpin structures were predicted based on the initial identification of the apical loop position. Primary hairpin stem length was determined by counting the number of bases within the stem starting at the first paired base after the apical loop and stopping at the basal unpaired region. Primary sequence motifs (CNNC, basal UG, apical UGU/UGUG^23^ and secondary structure motifs (e.g., apical loop size, internal bulge position, single stranded regions) were then annotated on each candidate hairpin. Comparisons were then made between WT and mutant structure annotations. A number of structural features were frequently impacted in mutant pri-miRNA hairpins including right bulge size (count of unpaired bases on the 5′ side of hairpin), left bulge size (count of unpaired bases on the 3′ side of hairpin), right bulge number (count of unpaired regions on the 5′ side of hairpin), left bulge number (count of unpaired regions on the 3′ side of hairpin), size of stem (length of the hairpin stem), loop size (count of unpaired bases within the loop), and loop number (count of predicted loops).

### miRNA Sensor and Biogenesis Assay

The miRNA gene reporter assay was constructed as previously described^26^. sgRNA (100 ng/μl), Cas9 (150 ng/μl) with or without the corresponding GFP-miR-24-MRE or GFP-Let-7-7-MREs (50 ng/μl) and mCherry (50 ng/μl) with Phenol Red were injected. 48 hpf embryos were imaged with a confocal microscope (SP5 Leica Microsystems) and captured using Leica application software suite. Mutant and wild type miRNA genome sequence were cloned as previously described^31^. Northern blots were performed using 3 micrograms of total RNA extracted from mCherry positive injected embryos and developed as reported previously in^26^.

## Additional Information

**How to cite this article**: Narayanan, A. *et al.* In vivo mutagenesis of miRNA gene families using a scalable multiplexed CRISPR/Cas9 nuclease system. *Sci. Rep.* **6**, 32386; doi: 10.1038/srep32386 (2016).

## Supplementary Material

Supplementary Information
