# Supplementary material for: In vivo mutagenesis of miRNA gene families using a scalable multiplexed CRISPR/Cas9 nuclease system
Source: Sci Rep. 2016 Aug 30;6:32386. doi: 10.1038/srep32386 (PMC5004112; doi:10.1038/srep32386)
Supplement: Supplementary Information [file srep32386-s1.pdf]

Supplementary information:

In vivo mutagenesis of miRNA gene families using a scalable multiplexed CRISPR/Cas9 nuclease system

Anand Narayanan, Guillermina Hill-Teran, Albertomaria Moro, Emma Ristori, Dionna M. Kasper, Christine Roden, Jun Lu, and Stefania Nicoli

Name	Chr location	st	Gene type	Mature ID	Sequence (ClustalW2 alg)	Sequence (MEMEalg)
miR-22a	chr 15: 24261942-24262027	-	intergenic	MIMAT0031942	AAGCUGCCAGCUGAAGAACUGU	
miR-25	chr 14: 205362-205443	-	intron	MIMAT0031946	CAUUGCACUUGUCUCGGUCUGA	
miR-187-1	chr 19: 35551006-35551094	+	intron	MIMAT0001274	UCGUGUCUUGUGUUGCAGCC	
miR-128-1	chr 22: 12504146-12504243	+	intron	MIMAT0031967	UCACAGUGAACCGGUCUCUUUU	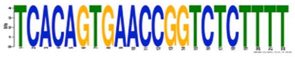
miR-128-2	chr 19: 44518214-44518336	+	intron	MIMAT0001824	UCACAGUGAACCGGUCUCUUUU	
					**********************	
miR-99-1	chr 15: 29144677-29144760	+	intron	MIMAT0001812	AACCCGUAGAUCCGAUCUUGUG	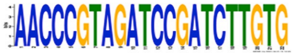
miR-99-2	chr 10: 39438831-39438910	-	intergenic	MIMAT0001812	AACCCGUAGAUCCGAUCUUGUG	
					**********************	
miR-92a-1	chr 1: 2806869-2806953	+	intergenic	MIMAT0031953	AGGUUGGGAUUGGUAGCAAUGCU	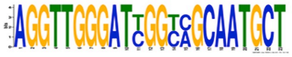
miR-92a-2	chr 9: 55420212-55420306	-	intergenic	MIMAT0031954	AGGUUGGGAUCGGCCGCAAUGCU	
					********** ** ********	
miR-23a-1	chr 22: 4997592-4997680	+	intergenic	MIMAT0001790	AUCACAUUGCCAGGGAUUUCCA	
miR-23a-2	chr 3: 13208734-13208831	+	intergenic	MIMAT0031944	AUCACAUUGCCAGGGAUUUCCA	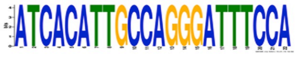
miR-23a-3	chr 2: 33618310-33618387	+	intergenic	MIMAT0031945	AUCACAUUGCCAGGGAUUUCCA	
miR-23a-4	chr 3: 13304718-13304815	-	intergenic	MIMAT0031944	AUCACAUUGCCAGGGAUUUCCA	
					**********************	
miR-24-1	chr 10: 15989409-15989488	+	exon	MIMAT0001792	UGGCUCAGUUCAGCAGGAACAG	
miR-24-2	chr 3: 13215487-13215568	+	intergenic	MIMAT0001792	UGGCUCAGUUCAGCAGGAACAG	
miR-24-3	chr 8: 31010534-31010675	-	exon	MIMAT0001792	UGGCUCAGUUCAGCAGGAACAG	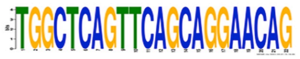
miR-24-4	chr 22: 5011864-5011953	+	intergenic	MIMAT0001792	UGGCUCAGUUCAGCAGGAACAG	
miR-24-5	chr 3: 13298295-13298376	-	intergenic	MIMAT0001792	UGGCUCAGUUCAGCAGGAACAG	
					**********************	
miR-125a-1	chr 16: 27137452-27137534	+	intergenic	MIMAT0001820	UCCCUGAGACCCUUAACCUGUG-	
miR-125a-2	chr 19: 10066308-10066414	+	intergenic	MIMAT0001820	UCCCUGAGACCCUUAACCUGUG-	
miR-125b-1	chr 15: 20409298-20409442	+	intergenic	MIMAT0001821	UCCCUGAGACCCU-AACUUGUGA	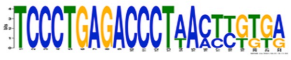
miR-125b-2	chr 5: 31637252-31637337	+	intergenic	MIMAT0001821	UCCCUGAGACCCU-AACUUGUGA	
miR-125b-3	chr 10: 39415823-39415937	-	intergenic	MIMAT0001821	UCCCUGAGACCCU-AACUUGUGA	
miR-125c	chr 15: 29150062-29150146	+	intron	MIMAT0001822	UCCCUGAGACCCU-AACUCGUGA	
					************* *** ***	
let7a-1	chr 11: 28380072-28380160	-	intergenic	MIMAT0001759	UGAGGUAGUAGGUUGUAUAGUU	
let7a-2	chr 15: 20399518-20399601	+	intergenic	MIMAT0001759	UGAGGUAGUAGGUUGUAUAGUU	
let7a-3	chr 4: 17722339-17722445	-	intergenic	MIMAT0001759	UGAGGUAGUAGGUUGUAUAGUU	
let7a-4	chr 5: 31628942-31629034	+	intergenic	MIMAT0001759	UGAGGUAGUAGGUUGUAUAGUU	
let7a-5	chr 23: 5478470-5478593	-	intergenic	MIMAT0001759	UGAGGUAGUAGGUUGUAUAGUU	
let7a-6	chr 6: 54461616-54461744	-	intergenic	MIMAT0001759	UGAGGUAGUAGGUUGUAUAGUU	
let7b-1	chr 4: 17721332-17721425	-	intergenic	MIMAT0001760	UGAGGUAGUAGGUUGUGUGGUU	
let7c-1	chr 15: 29144917-29145023	+	intron	MIMAT0001761	UGAGGUAGUAGGUUGUAUGGUU	
let7c-2	chr 10: 39436596-39436687	-	intergenic	MIMAT0001761	UGAGGUAGUAGGUUGUAUGGUU	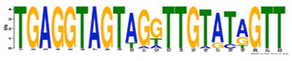
let7d-1	chr 16: 27120415-27120511	+	intergenic	MIMAT0001762	UGAGGUAGUUGGUUGUAUGGUU	
let7d-2	chr 19: 10052393-10052480	+	intergenic	MIMAT0001762	UGAGGUAGUUGGUUGUAUGGUU	
let7e	chr 23: 5478692-5478791	-	intergenic	MIMAT0001763	UGAGGUAGUAGAUUGAAUAGUU	
let7f	chr 11: 28379857-28379972	-	intergenic	MIMAT0001764	UGAGGUAGUAGAUUGUAUAGUU	
let7g-1	chr 23: 18553174-18553269	-	intergenic	MIMAT0001765	UGAGGUAGUAGUUUGUAUAGUU	
let7g-2	chr 23: 28779253-28779349	+	intron	MIMAT0001765	UGAGGUAGUAGUUUGUAUAGUU	
let7h	chr 23: 28779557-28779670	+	intron	MIMAT0001766	UGAGGUAGUAAGUUGUGUUGUU	
let7i	chr 25: 1688203-1688297	-	intergenic	MIMAT0001767	UGAGGUAGUAGUUUGUGCUGUU	
let7j	chr 6: 41385655-41385737	+	intron	MIMAT0003015	UGAGGUAGUUGUUUGUACAGUU	
					********* *** ***	

Supplementary Figure 1

Summary table for the zebrafish miRNA families/duplicates analyzed. The miRNAs are grouped based on the sequence similarity of the mature miRNAs. For each family a sequence alignment was performed using ClustalW (Larkin, Blackshields et al. 2007) and MEME programs (Bailey, Boden et al. 2009). Identical sequences between miRNA family members are shown in red.

		gRNA target sequence (X_{20})		Genotype	
		gRNA (1)	gRNA (2)	Primer-For 5'-3'	Primer-Rev 5'-3'
M2	miR-22-a	TGCCAGCTGAAGAACTGTTG	GACCTGCAGCAGTTCTTCAC	CAAATTAAGGGTTGAGCTGACAA	ACAGAATATATCACTTCCTGATCCC
	miR-25	TGCCAGCTGAAGAACTGTTG	GACCTGCAGCAGTTCTTCAC	CCGTGACGACTGTTGTGT	CACCTTCAGCACTCTTCTTCT
	miR-187-1	GTGTCTTGTGTTGCAGCCAG	GGGGCTGCAACACAGGACAT	CCTTACACTCCATCTGTGTCTC	AACCTTTGCTTTGCACTTATCA
	miR-187-2	GTGTCTTGTGTTGCAGCCAG	GGGGCTGCAACACAGGACAT	ACACTCCATCTGTGTCTCTTTC	CCTCTCAGCAGAAGATAATGGG
M4	miR-128-1	TAGGCTTTCTCACAGTGAAC	AGTGCTGGGAGACGGGGCC	CAGTTCTCTCACTCGACTCAAC	GAGAGTCCTCATAAACCACCAA
	miR-128-2	CCACTCGTCTCACAGTGAAC	GTCTGTCAGTAGTAGGACAG	GTGCTTGTGAATGGCTGTATTT	TCCACACACTGAACCCATAAG
	miR-30-b	TCAGCTGTGAGCTGCAGACG	TGCAGACGAGGCTGGGCGGA	GTCGTGAGTGTTGTGTGGTAAA	AGTCGCTGTGCTCTTGAAATC
	miR-30-c	AAACATCCTACACTCTCAGC	GCTGGAGCGCAGCCGAGGCC	ATGACCTGTATCATAGGCAGTTT	GGTTGGAAGGACAGACAGATAG
	miR-30-d	CGACTGGAAGCTGTGCTACG	GTTCTGTCGCCTTGTATTAC	CAGCAAACCCACCACATAGA	GCCACACCTGTCAACTCTTTA
	miR-92-a-1	GCACTTGTCCCGGCCTGTAA	CTTTCTGCGCAGGTTGGGAT	TGTGGCAGCACACTTCTTA	ATTTGTGGGGTACAATCTC
	miR-92-a-2	TGGAAGTATTGCACTTGTCC	GCATCCCTTTCTTTGCAGGT	AAGTGCTACAACAGCAGAGAG	GCTGAGATTGTCGGACGTATT
	miR-99-1	CGATCTTGTGATAAGTTTGA	TCTATGGGTCTCTGTCTCTG	GTGAAGGTCATACGCTGATGTA	CTGTGGACAAGTCTGAGCAA
	miR-99-2	AACCCGTAGATCCGATCTTG	CAACCCAAGCTCGATTCTGT	GTTCCTCTGTGCACTGTTAATG	GGCAATGTTTCTGTGGTCTATC
M10	miR-23-a-1	ATCATAAAATCACATTGCCA	GATTCCTGGCAGAGTGATTT	GCTTTGACCTGGTTTGGATAAG	GGTGGAGCAAGTACGATAACA
	miR-23-a-2	CTGCCGGCCAGGGGAATTCC	ACATTGCCAGGGATTTCCAA	GTGCTGTGCCAACCTTATATTG	AGACAGTCGGCATTCCTTTAG
	miR-23-a-4	ATGACTGAATCACATTGCCA	ATGACTGAATCACATTGCCA	GAGACAGTCGGCATTCCTTT	TTCGGACAGATATAGCCGTGT
	miR-23-a-3	ATCATAAAATCACATTGCCA	GATTCCTGGCAGAGTGATTT	GAGAAGCCAGGGTCATGTAAA	GACCACTTCATCCATCAGACTC
	miR-24-1	CTCAGAAGGCACCGGAGCTC	TATCAGTTGTAGTAAATCAC	AAGCCCATGACCAAGGTAAG	AGCCCTCCTGGTTCATTTAAG
	miR-24-2	GTTCAGCAGGAACAGGGGCC	ATTATCAGCACAGCAGGCAC	GTGCCCTCTTTGTCTCTTCTT	GTGAAGGTGAAGCGTCCAG
	miR-24-3	ATCAGCTCAGTAGGCACGGG	GTTCAGCAGGAACAGGAGTC	TTGCACGGGCCGTATTTA	TCCTCCATGAAGGGATTTGTC
	miR-24-4	CCAGTTTAGCAGGCACAAGT	TCAGTATGTTGATTTAGTGC	CTGGTGTCGATATAGGCTCTTATG	ATCCTCGATGCCTTGACTTG
	miR-24-5	GTTCAGCAGGAACAGGGGCC	ATTATCAGCACAGCAGGCAC	TTCTCCCTCTGTCTGTGTCT	GTGAAGCGTCCAGGTTCATA
	miR-125-a-1	CCTGAGACCCTTAACCTGTG	CAGGTGAGGTCCTCAGGAAC	CCTGACAGTCTGAACAGCATAG	GATTGACGTTTGGGGATGACG
	miR-125-a-2	GTGATGTCTTCCAAGGTCAC	GACACAGACATACATCGATT	CGAGCCAGAAGTGTGAGTTT	TGGTTGAGGACCAATGGTTTAG
	miR-125-b-1	ACGTTTTCCTGTTATGTGCA	TTAGGTTCTTGGGAGCTGAG	GGTTGCACTTGTTGTGTCATAG	GCCGCAATTTCTTCAATCCC
	miR-125-b-2	ACGTTTTCCTGTTATGTGCA	GGGTTCTCGGGAGCTGTGAG	GGCGTGTGTGTGTGTAACTA	AGTGTCCTGACTCACAGTCTAA
	miR-125-b-3	CAGGTTAAGCTCTTGGGACC	AAGTTAGGGTCTCAGGGACC	CATATCTCTCACAGACATCCATCC	CAACAAAGACTCGACGGACA
M24	let-7a-1	GACGGTGGGATGAGGTAGT	TTTTAGGGTCACACCCACAC	ACTACCTCACACTGCACAAG	TAAATCCTAACATGGCGGGC
	let-7a-2	GCCCCCAGGCTGAGGTAGT	ATAGTTTAGAATAACATCAC	CTACTGATCAGGGCCTCGTT	CGGAAATCAGTGTTCGCTCC
	let-7a-3	ACTTACTGTCTTTCCCGAAG		TCAAGCATAAAACATTGCACACA	AGGCAGGTGTTTCTCTGACA
	let-7a-4	ATGTCTCGGGATGAGGTAGT		GACACCTGCTAGTGCTAGTG	GGTGGAAACACTTGAAATGCTC
	let-7a-5	TCTGGACAAGGTGAGGTAGT		GCAGTGTAAACCAGCCACAT	GACAGCCAAATCATCCGCAA
	let-7a-6	TTTCTTCAAGGTGAGGTAGT		AGCTGACACCTATGATTGGTTT	AAATGCCAAATGACCCAGCC
	let-7b	TCGGACAGGGTGAGGTAGT		TTCATGCAACTTTAAATGGCTCA	AATTGTTCAAAGTTGCCAGGAG
	let-7c-1	TGCATCCAGGCTGAGGTAGT	GAGTGTGTGCATCCAGGCTG	TTTCCCTTTGCTTGTCAGGC	TCTCTGACATACGCTCAGTTGA
	let-7c-2			CCGGTGATGGACCATTTGTA	CCTGTCTGCCTATCCACTTTAG
	Let-7d-1	GCGTTGCGGTGTGAGGTAGT	GTGTGAGGTAGTTGGTTGTA	CTAAGGTCCTCAGCAGTCTTTC	CCCACTGTATCTAATGTAACCTCTC
	Let-7-d-2	CGCTGCAGGCTGAGGTAGT	GTTTTGCATCATAATCAGCC	ACACCTGCTAGTGCTAGTG	GGTGGAAACACTTGAAATGCTC
	let-7e	ACAATCTACTGTCTTTCCTA		CGCTTTGTCCGTGTTGTTTT	CTCAAGCCATTCTGTGAAGTCC
	let-7f	GTAGTAGATTGTATAGTTGT		GAGCGCTTCATATTATCCAGCA	CAAGCTACAGAGTTTCATCCTGT
	let-7g-1	AGGTAGTAGTTTGTATAGTT	GTTTGGGATCACACCAGATC	CCACAAAAGCCCTGTCTGTG	CAGCCTTTCACTGTGGGATT
	let-7g-2	GTAGTAGTTTGTATAGTTTT	ACAGTCTACTGTCTTTCCCA	AGGTGAGAGCACACTAAATG	TGAGTAGTTGCCTAGAAACAAT
	let-7h	GTAGTAAGTTGTGTTGTTGT		ACATGGCTTGCTTTTATCGC	GGCCTGTTCTCCTTCTGACA
	let-7i	GAGGTAGTAGTTTGTGCTGT		AGCGCACGTTTACAAAGCTA	GCTCACGAGTACTACGACCA
	let-7j	TAGTTGTTTGTACAGTTTTT		AGTGTATGCATCCGTTTCTATGT	GTACTGTATACCATGTCAGATGAGTG

Supplementary Table 1. Table reporting the sequences of all gRNAs used. Primers for the genotype of each miRNA locus are also included.
